# Multiclass malaria parasite recognition based on transformer models and a generative adversarial network

**DOI:** 10.1038/s41598-023-44297-y

**Published:** 2023-10-10

**Authors:** Dianhuan Tan, Xianghui Liang

**Affiliations:** Department of Clinical Laboratory, Xiangya Hospital, Central South University, No.87 Xiangya Road, Kaifu District, Changsha, 410008 Hunan China

**Keywords:** Biotechnology, Cell biology, Microbiology

## Abstract

Malaria is an extremely infectious disease and a main cause of death worldwide. Microscopic examination of thin slide serves as a common method for the diagnosis of malaria. Meanwhile, the transformer models have gained increasing popularity in many regions, such as computer vision and natural language processing. Transformers also offer lots of advantages in classification task, such as Fine-grained Feature Extraction, Attention Mechanism etc. In this article, we propose to assist the medical professionals by developing an effective framework based on transformer models and a generative adversarial network for multi-class plasmodium classification and malaria diagnosis. The Generative Adversarial Network is employed to generate extended training samples from multiclass cell images, with the aim of enhancing the robustness of the resulting model. We aim to optimize plasmodium classification to achieve an exact balance of high accuracy and low resource consumption. A comprehensive comparison of the transformer models to the state-of-the-art methods proves their efficiency in the classification of malaria parasite through thin blood smear microscopic images. Based on our findings, the Swin Transformer model and MobileVit outperform the baseline architectures in terms of precision, recall, F1-score, specificity, and FPR on test set (the data was divided into train: validation: test splits). It is evident that the Swin Transformer achieves superior detection performance (up to 99.8% accuracy), while MobileViT demonstrates lower memory usage and shorter inference times. High accuracy empowers healthcare professionals to conduct precise diagnoses, while low memory usage and short inference times enable the deployment of predictive models on edge devices with limited computational and memory resources.

## Introduction

Malaria, a mosquito-borne disease transmitted by various mosquito species, poses a significant global threat to both humans and other creatures. Malaria is caused by plasmodium, which is one of most pronounced deadly protozoan parasites. It harms the red blood cells, and eventually causes serious disease^1^. According to the world malaria report in 2022^2^, more than 619,000 deaths were reported in 2021, most of them are children. Malaria prevention and control efforts are burdened with numerous challenges, apart from the existing significant COVID-19 interruption and other healthcare system hurdles. These additional challenges include prolonged humanitarian crises, insufficient funding from donors, and the potential impact of climate change on the spread of the disease. Reference^3^ revealed that Malaria infection remains one of the main killers of children aged 6–59 months. Reference^4^ showed that the number of confirmed malaria cases in children under 5 years increases with increase in rainfall and the number of tested cases reduces with increase in temperature. India contributes to 1.7% of the global malaria cases, with a significant proportion of fatalities occurring in children under the age of five within the country. The mosquito responsible for malaria transmission is prevalent in regions spanning America, Africa, Asia, and Latin America, primarily affecting areas lacking the necessary resources for prevention. Africa, in particular, bears a staggering 90% of the global malaria-related mortality burden, with 68% of Ethiopia's population and 97% of the Democratic Republic of the Congo's population residing in high-risk malaria infection zones^5,6^. Severe malaria is primarily attributed to P. falciparum, commonly known as falciparum malaria, with symptoms typically manifesting 9–30 days after infection. Research conducted by Ref.^7^ reveals that the majority of malaria-related deaths are caused by P. falciparum (p.f), whereas P. vivax(p.v), P. ovale(p.o), and P. malariae(p.m) generally result in milder forms of the disease.

The reliable diagnosis of malaria faces a significant challenge due to the presence of P. falciparum parasites with Pfhrp2/3(specific gene found in the Plasmodium falciparum parasite) gene deletions, which undermines the effectiveness of malaria diagnostic tests. To address this issue, a diverse range of diagnostic approaches is required. While methods that employ polymerase chain reaction (PCR) for malaria parasite DNA detection or utilize PCA Model for RNA-Seq Malaria Vector Data Classification^8^ have been developed, their widespread application in malaria-endemic areas has been hindered by their high cost and complexity. Consequently, the standard method for malaria diagnosis remains the microscopic analysis of stained blood slides^9^. While slide examination is an economical and straightforward technique, it presents significant time and difficulty challenges. This is due to the fact that only a small portion of the slides is visible through the microscope, and small-sized parasites are often sparsely distributed. As a result, a comprehensive examination typically consumes approximately 10–15 min. It is susceptible to human errors, and the scarcity of experts further exacerbates the challenge. Therefore, there is a pressing need for automated diagnosis methods aimed at detecting malaria parasite-infected blood cells through the analysis of microscopic blood cell images. For this reason, key research and development challenges involve the limited sensitivity of tools for detecting non-P. falciparum species and the necessity for a broader range of diagnostic applications^2^. Given their increasing precision and resilience in microscopic analysis, deep learning models offer a promising solution. In this paper, we propose the design of an efficient deep learning framework for the noninvasive classification of multiple Plasmodium species and malaria diagnosis.

Deep learning has a large number of applications in the image processing field. Due to their high speed, accuracy, flexibility and low cost^1^, deep learning models has gained huge popularity and has been widely applied in CT-scans^10^, MRI^11^ and various microscopicimage analyses^12–14^, such as microscopic examination of protozoan including plasmodium classification for thin smears. Furthermore, it's worth noting that Transformer models have also been employed in various classification tasks recently^15^. Reference^16^ evaluated their proposed classifier on 27,560 samples, achieving an accuracy of 98.37% on average using tenfold cross-validation.

Current works in malaria classification primarily focus on determining whether blood cells are infected or not. To achieve this objective, Diker^17^ introduced an optimized Residual Convolutional Neural Network that utilizes the Bayesian method to classify malaria cell images as infected or non-infected. In the classification of malaria cell images, the addition of Neighborhood Components Analysis (NCA) has been observed to enhance the performance of classifiers. Ufuktepe et al.^18^ presented a channel-wise feature pyramid network specifically designed for medical applications, which utilizes the green channel of input images to detect parasite cells and classify them as infected or non-infected. They expanded their approach by introducing an additional class for P. falciparum and training the network to categorize different types of infected parasites. Molina et al.^19^ developed a convolutional neural network approach capable of distinguishing between all stages of malaria-infected red blood cells. They utilized transfer learning with the VGG-16 architecture for their experiments. Yang F et al.^20^ proposed the first system that combines deep learning models and image processing techniques for detecting malaria parasites in thick smears, and they successfully implemented these models on smartphones. Reference^21^ designed a segmentation technique to detect parasite cells of malaria in thin blood smear images using edge-based segmentation. The proposed method achieved high accuracy, SE, SP, PVP, and PVN values. Results in this study indicated that the edge-based segmentation technique is a promising technique to facilitate the malaria classification. Reference^22^ introduced a novel approach known as Deep Cycle Transfer Learning (DCTL) for parasite detection. DCTL offers an effective solution to circumvent the laborious process of data annotation by transferring macro-level shape knowledge from human experts, thereby substituting the need for micro-level shape knowledge of parasites. Reference^23^ introduced an innovative deep learning approach that is inherently geometry-aware. This method leveraged a geometric-feature spectrum to effectively detect the presence of host nucleus, Toxoplasma, Trypanosome, and Babesia using an architecture based on ExtremeNet. Notably, this approach achieved a commendable feat by ensuring accurate detection without any instances of misdiagnosis. It stands apart from conventional unsupervised learning methods due to its unique design and capabilities. In Ref.^24^, an automated and quantitative analysis of Babesia-infected erythrocytes was conducted utilizing DenseNet. This approach effectively circumvented the quantitative analysis errors that often arise from manual microscopy. Moreover, the utilization of Integrated Gradient as an interpretability tool for the model shed light on the primary contributors to false positives, which were identified as cell boundaries, precipitate, and rouleaux formations.

After the Plasmodium parasite infects an animal through a mosquito bite, it undergoes two distinct life stages within its host. The first stage is an asymptomatic liver stage, followed by a blood stage where clinical symptoms of malaria manifest. Additionally, cells infected with the malaria parasite progress through various life stages during the development of the disease, including gametocyte, ring, and schizont stages. Recognizing the significance of these lifecycle stages, other researchers have focused their efforts on understanding and classifying Plasmodium at different stages. Salam et al.^25^ devised a two-stage algorithm that utilizes the filter method for feature ranking and incremental feature selection technique for analysis. They trained various machine learning models, such as SVM, k-NN, and ANN, on a dataset to evaluate their performance. To achieve improved classification performance, they developed a hybrid classifier that combines three different classifiers, surpassing the performance of individual classifiers. Arshad et al.^26^ proposed a deep learning-based two-stage approach for the multi-class classification of P. vivax lifecycle stages. They employed the Unet architecture to segment cells from images captured by a microscopic camera and selected the ResNet network for single-stage multi-class classification.

After analysis of the literature concerning the diagnosis of malaria disease, it was evident that there exists a pressing need to address the comprehensive classification of multiclass malaria species, which is unexplored. It is noteworthy that the majority of the existing research has been primarily centered around binary classification tasks. In addition to the requirement for infected cells classification, inadequate computing resources in least developed countries pose a special challenge. To solve this issue, we present the performance of several models including VGG-19, Swin Transformer and MobileViT. The aim of this research paper is to systematically select the best model for the purpose of malaria case finding in regions where malaria has remained endemic. To handle real-world situation, a multi-labelled dataset is targeted. Data was collected from Hunan province in the South of China.

In this paper, we chosen two convolutional networks and three transformer-based models including Swin Transformer, Vision Transformer and MobileViT. As a general-purpose and effective model for computer vision, Swin Transformer is a model commonly used for medical image classification. Also, we present the performance of MobileViT taking resource and energy consumption into consideration. Besides, we chosen WGAN-GP to augment our dataset.

The primary objective of this paper is to present a robust solution for malaria diagnosis through microscopic examination, leveraging deep learning models. In the following sections, we will provide details regarding dataset collection and our approach to training the neural networks.

The rest of the paper is structured as follows. Section “Dataset” elucidates the process of dataset acquisition involved in the proposed work. Section “Methods” demonstrates the methodology employed in the research to attain its objectives. Section “Results” describes the results of experiments done on the dataset using the proposed methodology. Section “Discussions” is a comprehensive discussion of the proposed work and potential avenues for future research. Section “Conclusion” outlines the limitations of the research.

## Dataset

**Figure 1:**
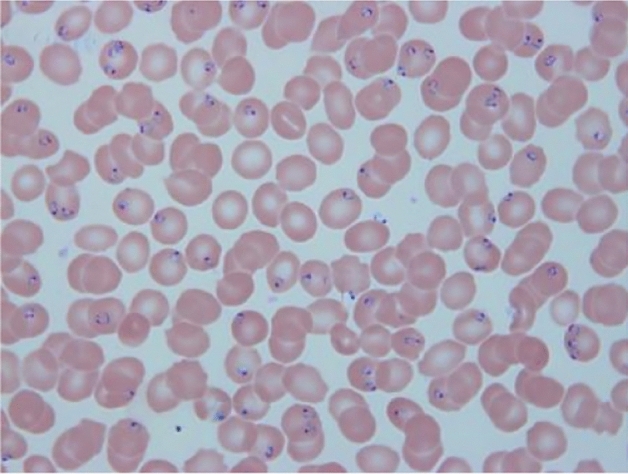
Microscopic p.f ring image directly from smears.

**Figure 2:**
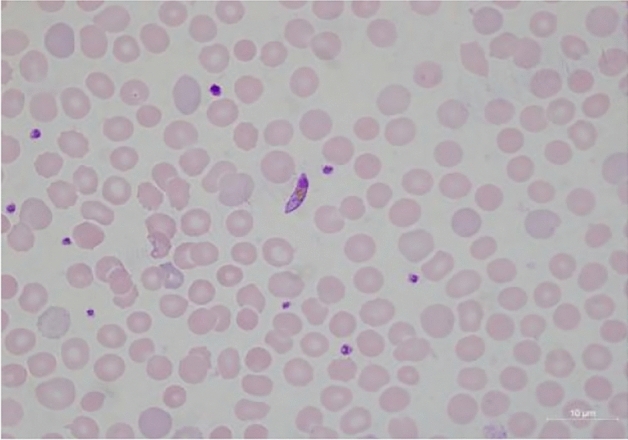
Microscopic p.f gametocyte image directly from smears.

In this paper, our dataset, which consists of in total 390 blood smear images as shown in Figs. 1 and 2, is taken from approximately one hundred patients. Those images were collected from thin blood smears^26^ from malaria-infected patients in Changsha city of Hunan province. Blood smears were meticulously prepared by spreading a thin layer of blood onto glass slides and leaving them to air-dry. Subsequently, to ensure fixation, methanol was applied. Giemsa solution was then applied to stain the blood smear, with an incubation period of 10 to 15 min. Finally, to secure the integrity of the specimens, cover-slips were permanently affixed to the slides. It is noteworthy that these thin-film blood slides, constituting the dataset, were crafted by skilled professionals within laboratory settings. The microscopic images were acquired using an high-resolution microscope at 100 × objective magnification, with annotations and captures overseen by an expert haematologist. The dataset statistics are shown in Table 1.

**Table 1:** Cells distribution.

Classes	Number of cells
p.f (P. falciparum)	187
p.m (P. vivax)	113
p.o (P. ovale)	93
p.v (P. malariae)	168
PLT (platelet)	457
WBC (white blood cell)	300
RBC (red blood cell)	300

Single cell images annotated from original blood smears microscopic images (for example, Figs. 1 and 2).

The cells were carefully categorized into different classes, encompassing platelets, red blood cells, white blood cells, as well as four species of the malaria plasmodium, namely P. falciparum(p.f), whereas P. vivax(p.v), P. ovale(p.o), and P. malariae(p.m). Within our collection of microscopic images, each image included more than one hundred cells in total, with a predominant presence of healthy red blood cells. In our proposed methodology, we deliberately select only 300 of them for the balance across all classification classes.

## Methods

For the multi-classification of malaria parasite lifecycle, the Swin Transformer network has been designed using a dataset of microscopic thin blood smear images. The system configuration used for experiments is Intel core i3-10,100 processor with Nvidia 1650 GPU on Windows 11 operating system with 64GB memory. The models are trained in Python 3.6.13 enviroment using pytorch 1.13.1. The microsocpic image dataset used as input data for model training and valiadation is augmented with various techniques to improve the model performance as well as help train robust and accurate machine-learning models. The source code for WGAN-GP can be accessed through the following link: https://github.com/igul222/improved_wgan_training^27^. Additionally, the code for MobileViT is available at: https://github.com/micronDLA/MobileViTv3^28^.

**Figure 3:**
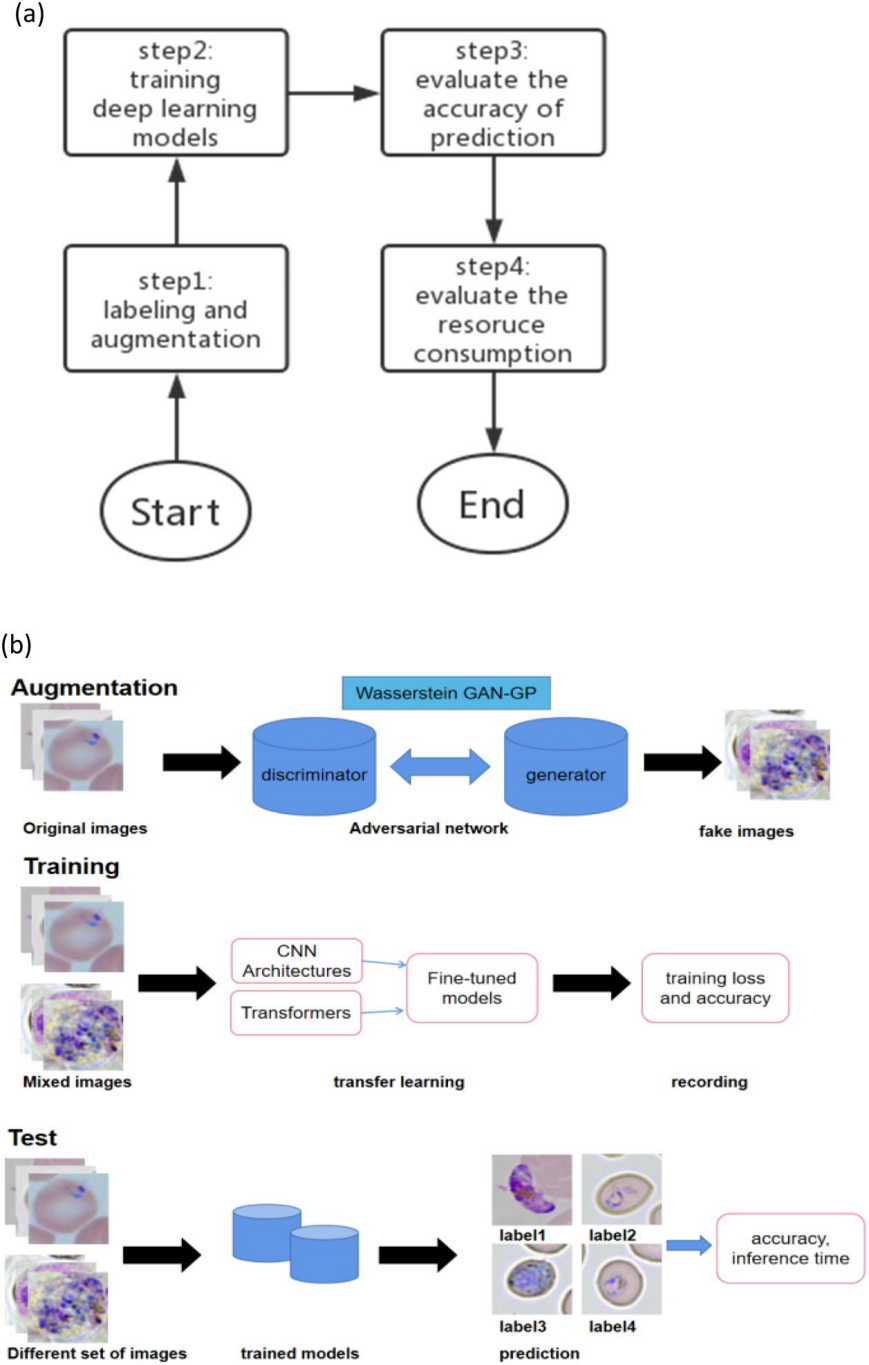
(**a**) presents the flowchart for steps by which we conducted our experiment. (**b**) illustrates the malaria analysis pipelines employed in (**a**), the augmentation pipeline was employed in step1, training pipeline in step2 and step3, test pipeline in step3 and step4.

The framework for multiclass lifecycle malaria analysis is presented in Fig. 3. It contains four steps where three pipelines exist.

Following the initial data acquisition from blood smears in Section “Dataset”, this section proceeds to delineate subsequent steps. Section “Data labelling and augmentation” introduces the techniques employed for data labeling and augmentation, aimed at expanding the dataset's size and achieving a more balanced representation to mimic real-world scenarios. In Section “GAN architecture”, we delve into the details of the GAN (Generative Adversarial Network) architecture adopted in this research. Section “Swin Transformer” provides an overview of the Swin-Transformer architecture, while Sects. “Window-based self-attention” and “Shifted window self-attention” elucidate its advantages and how they contribute to enhanced accuracy. Lastly, Sect. “Light-weight vision transformer: MobileViT” introduces MobileViT, another transformer model distinguished by its lightweight and high efficiency.

### Data labelling and augmentation

**Figure 4:**
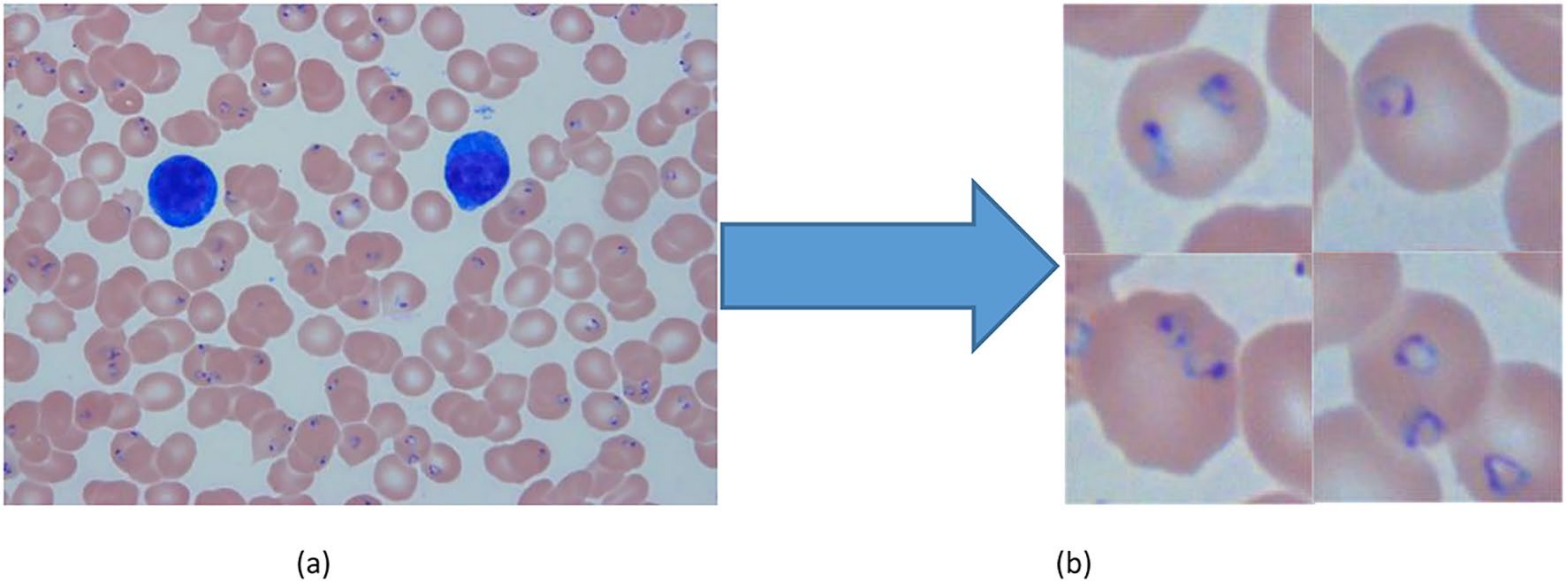
Single cell from a original image (**a**), four rings (**b**) are selected and labeled.

The dataset prepared for model training were single cells images labeled from microscopic smear images captured by an microsope at 1000 × magnification. Figure 4 show a example we labeled cell images.

**Figure 5:**
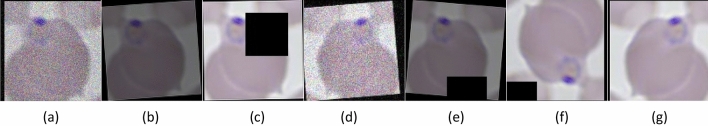
Common techniques for data augmentation: add noise (**a**) change light (**b**), cut out (**c**), rotation (**d**), cropping (**e**), shifting (**f**), flipping (**g**).

After obtaining a sufficient number of images for classifier training, a notable issue emerged: our dataset exhibited an overrepresentation of healthy red blood cells compared to other classes, resulting in a pronounced imbalance and bias. This disparity, where the healthy class was disproportionately represented, prompted us to implement diverse augmentation techniques as a corrective measure. In addressing this challenge, we introduced practical approaches, including random flipping, shearing, and rotation at various angles, as illustrated in Fig. 5. These transformations effectively contributed to the creation of a more balanced dataset, mitigating the issue of class imbalance and bias.

The major approaches for Data Augmentation include: (1) geometric transformation, which reduce the difference in position, scale and perspective. (2) color adjustment that eliminate the difference caused by sunlight, luminance and color. (3) kernel filters, which improve the ability of models to process blur images. (4) Random erasing, a techniques to ensure the architecture to properly handle incomplete image and pay more attention to big picture, in stead of part of it. (5) Generative Adversarial Network(GAN)^29^ where a generator works to find a artifical distribution similar to real distribution.

The GANs have many applications in medical image processing. Zhang et al.^12^ used a pixel2pixel(a type of deep learning model architecture used for image-to-image translation tasks) GAN with V-Net(a network based on convolutional neural networks, specifically designed to handle 3D data) as the generator to correct motion artifacts in the right coronary artery, confirming that GANs have the potential to provide a certain approach of removing motion artifacts in image processing. Besides, GANs performs well in image generation by studying a collection of training examples and learn the probability distribution that generated them. They offer more possibilities than ordinary CNNs(convolutional neural networks)^29^. The Wasserstein GAN(WGAN) introduces the Wasserstein distance and provide a reliable training process indicator that improves the quality of generated images^30^. The WGAN with a gradient penalty (WGAN-GP) is an improved version of the WGAN that allows for easier optimisation and more stable convergence compared to the original WGAN.

The training strategy of GANs is to define a game between two competing network. The objective of generator G is to generate counterfeit cell image samples, while discriminator D is tasked with discerning whether a given cell image sample is authentic, sourced from the training set, or counterfeit. These two models operate concurrently through an adversarial process. Formally, the game between the G and the D is the minimax objective:

1 $$\begin{array}{c}\mathrm{min\,max}\\ \mathrm{G\,D}\end{array} {\mathrm{ V}(\mathrm{G},\mathrm{D})=\mathrm{ E}}_{\mathrm{x}\sim {\mathrm{P}}_{\mathrm{r}}}\left[\mathrm{logD}\left(\mathrm{x}\right)\right]+{\mathrm{E}}_{\mathrm{x}\sim {\mathrm{P}}_{\mathrm{g}}}\left[\mathrm{log}\left(1-\mathrm{D}\left(\widetilde{\mathrm{x}}\right)\right)\right].$$

Or

2 $$\begin{array}{c}\mathrm{min\,max}\\ \mathrm{G\,D}\end{array}\mathrm{ V}\left(\mathrm{G},\mathrm{D}\right)={\mathrm{ E}}_{\mathrm{x}\sim {\mathrm{P}}_{\mathrm{r}}}\left[\mathrm{logD}\left(\mathrm{x}\right)\right]-{\mathrm{E}}_{\mathrm{x}\sim {\mathrm{P}}_{\mathrm{g}}}\left[\mathrm{logD}\left(\widetilde{\mathrm{x}}\right)\right],$$

where ${\mathrm{P}}_{\mathrm{r}}$ is the real data distribution(real cells images in this research) and ${\mathrm{P}}_{\mathrm{g}}$ is the fake data distribution (fake cells images generated by the generator G in this reasearch).

The fake data distribution ${\mathrm{P}}_{\mathrm{g}}$ is implictly defined by $\widetilde{\mathrm{x}}$=G(z) where the input z to the generator G is sampled from some simple noise distribution (such as a Isotropic Gaussian distribution).

The Eq. (2) is a modification of (1), designed to enhance computational performance. The reason may intuitively be that (2) have greater gradients early when D(x) near zero, making the model adjust its parameters steeply, although $\mathrm{log}(1-\mathrm{D}(\widetilde{\mathrm{x}}))$ and $\mathrm{logD}(\widetilde{\mathrm{x}})$ result in the same fixed point, where ${\mathrm{P}}_{\mathrm{g }}= {\mathrm{P}}_{\mathrm{r}}$ and $\mathrm{D}(\mathrm{x})=1/2$. (the G generate samples as real and the D is unable to discriminate whether a sample from the training set or a generated set)^31^.

To solve the problem of unstable training and mode collapse which is common in GANs^30^, propose WGAN and suggest that the Jensen-Shannon divergence which GANs frequently minimize are potentially not continuous with respect to the generator’s parameters, leading to difficulty in convergence. The WGAN uses the Earth-mover distance, which means the minimum distance to transform the distribution $\mathrm{p}$ to distribution q. It works to minimize the cost required to transform one probability distribution p into another q. The basic equation is as follows:

3 $$W[p,q]=\underset{\gamma \in \Pi [p,q]}{inf}\iint \gamma (x,y)d(x,y)dxdy$$

$W[p,q]$ is the Earth Mover's Distance between two probability distributions.

$\underset{\gamma \in \Pi [p,q]}{inf}$ indicates that we are searching for the minimum value among all possible ways to transport the mass from p to q. The goal is to find the optimal transport plan that minimizes the total cost or work.

$d(x,y)$ represents the amount of mass to be transported from x in distribution p to y in distribution q according to the optimal transport plan.

$\gamma (x,y)$ represents the distance associated with moving one unit of mass from x in distribution p to y in distribution q.

**Figure 6:**
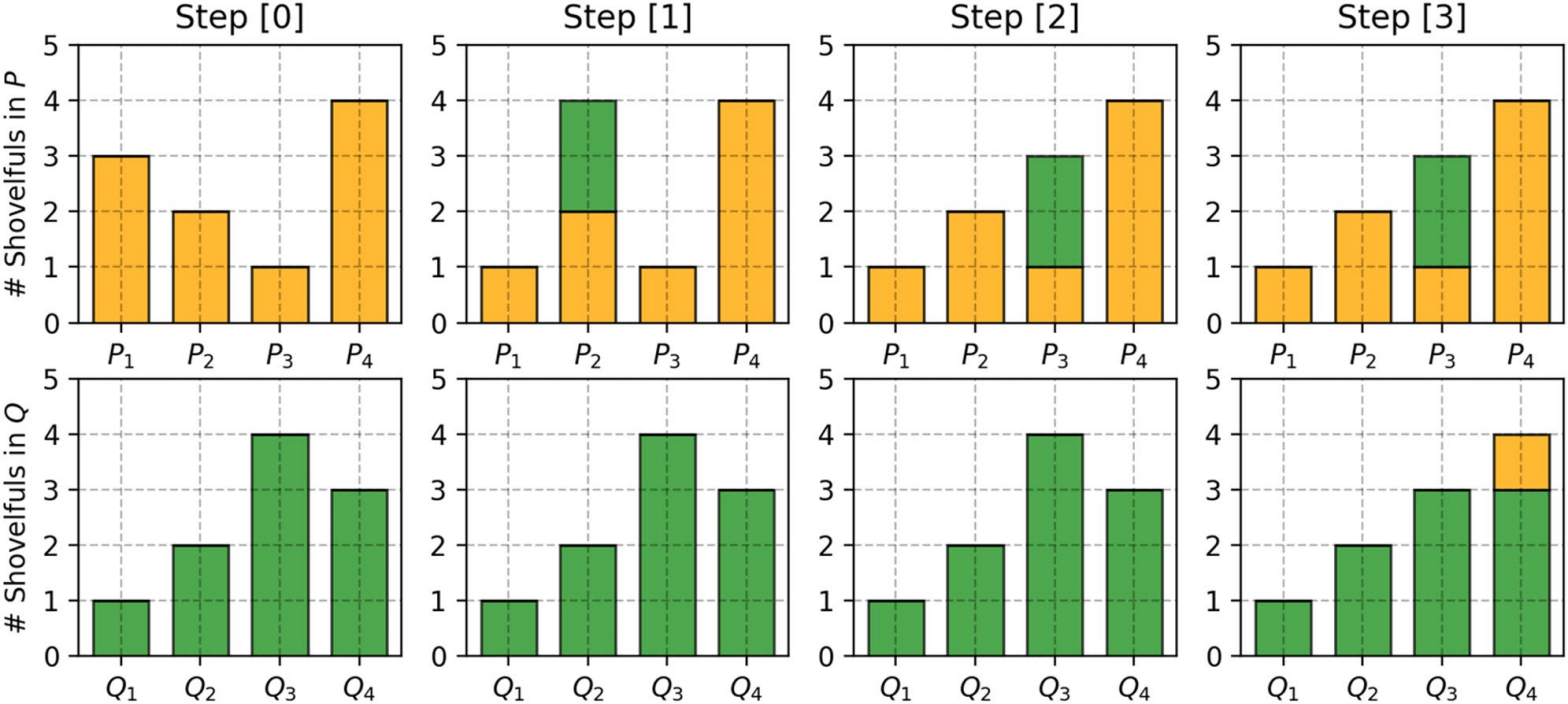
To change P to look like Q: (1) move 2 shovelfuls from *P*_*1*_ to *P*_*2*_ so that (*P*_*1*_* ,Q*_*1*_) match up. (2) move 2 shovelfuls from *P*_*2*_ to *P*_*3*_ so that (*P*_*2*_*, Q*_*2*_) match up. (3) move 1 shovelfuls from *P*_*3*_ to *P*_*4*_ so that (*P*_*3*_* ,Q*_*3*_) and (*P*_*4*_* ,Q*_*4*_) match up. Labeling the cost to pay to make (*P*_*i*_*,Q*_*i*_) match as $\updelta$_i_, we have $\updelta$_i +1_ = $\updelta$_i_ + *P*_*i*_* − Q*_*i*_*.* $\mathrm{In \,this\, example\, \delta }$_0_ = 0, $\updelta$_1_ = $\updelta$_0_ + 3 − 1 = 2, $\updelta$_2_ = 2 + 2 − 2 = 2, $\updelta$_3_ = 2 + 1 − 4 = -1, $\updelta$_4_ = − 1 + 4—3 = 0, $\mathrm{W}=\sum \left|\mathrm{\delta i}\right|=5$.

A simple discrete case of this equation could be illustrated as shown in Fig. 6^32^.

However, in continuous probability space, $x$ as the starting point and $y$ as the destination, the total amount of dirt moved is $d(x,y)$ and the travelling distance is $\gamma (x,y)$ and thus the cost is $\gamma (x,y)d(x,y)$.

One of the most popular Generative Adversarial Networks is improved Wasserstein generative adversarial network with a gradient penalty (WGAN-GP)^27,33^. We used WGAN-GP to synthesize cells images. Compared to GAN, the WGAN was proposed in 2017 to solve the issue of gradient disappearance, gradient instability and collapse mode. The WGAN-GP improved on the basis of GAN, delivering better performance in gradient stability and image generation. The most common problems for a Generative Adeversial Network are gradient disapperance and model collapse. Thus, WGAN-GP used a loss founction directly constraining the gradient norm of the critic’s output with respect to its input. To circumvent tractability issues, it enforce a soft version of the constraint with a penalty on the gradient norm for random samples. The least-squares loss in the training content discriminator and training generator was defined as follows:

4 $$L{\mkern 1mu} = {\mkern 1mu} \mathop {\mathbb{E}}\limits_{{\tilde{x}\sim {\mathbb{P}}_{g} }} \left[ {D\left( {\tilde{x}} \right)} \right] - \mathop {\mathbb{E}}\limits_{{x\sim {\mathbb{P}}_{r} }} \left[ {D\left( x \right)} \right] + \lambda \mathop {\mathbb{E}}\limits_{{\hat{x}\sim {\mathbb{P}}_{x} }} \left[ {\left( {\left\| {\nabla_{{\hat{x}}} D\left( {\hat{x}} \right)} \right\|_{2} - 1} \right)^{2} } \right]$$

The total loss function in WGAN-GP combines the generator loss, discriminator loss, and the gradient penalty. The first two terms were explained in (2).

The gradient penalty is a crucial addition in WGAN-GP. Mathematically the third term in (4).

Where: λ is a hyperparameter that controls the importance of the gradient penalty. ∇ denotes the gradient operator. $\hat{x}$ is the input of the discriminator. $D(\hat{x})$ is the discriminator's output when applied to a random linear combination of real data and generated data.

The gradient penalty is added to enforce a Lipschitz constraint on the discriminator, which helps stabilize the training process. The gradient penalty term encourages the gradients of the discriminator to be close to 1 in order to prevent the vanishing gradient problem. It's computed as the norm of the gradients of the discriminator's output with respect to random samples taken along straight lines between real and generated data points.

### GAN architecture

**Figure 7:**
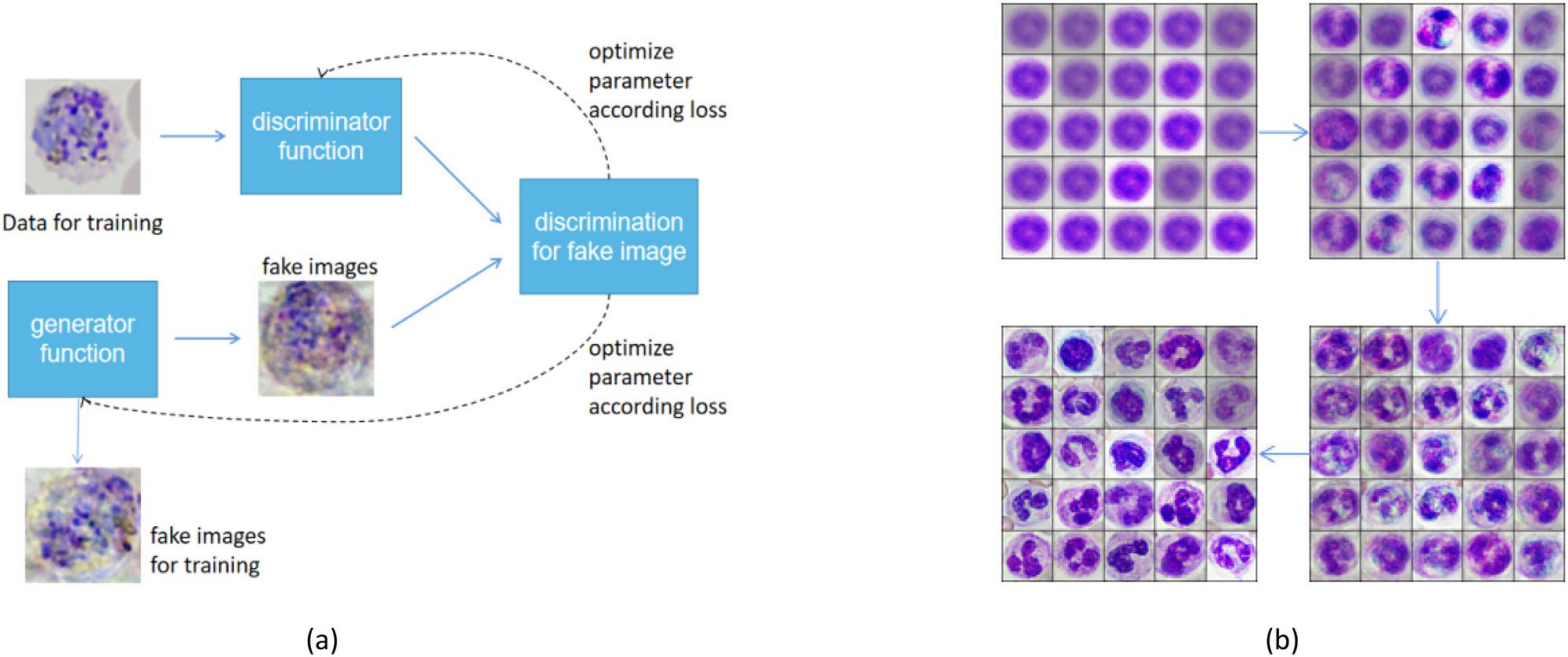
(**a**) training process (**b**) performance of generator.

In this paper, we employed a Wasserstein Generative Adversarial Network with Gradient Penalty (WGAN-GP), consisting of a generator and a discriminator. The training process is visually depicted in Fig. 7, where (a) delineates the training procedure of the WGAN-GP and the generation of cell images, while (b) provides insight into the generator's performance throughout the training process.

The discriminator was a CNN with 6 non-linear steps, in which first 4 layers were convolutional layers, and the activation function is leaky rectified linear unit (ReLU). A discrimitor was set to enhance the generaliztion of the generator. The discrimitor consisted of 5 non-linear blocks which each containing a convolution layer, leaky ReLU and average pool. The princinple of leaky ReLU is to upsample by applying a 2D transposed convolution operator over an input image composed of several input planes. This module can be seen as the gradient of Conv2d with respect to its input. The discriminator takes microscopic blood cell images of size 128 * 128 * 3 as input. It is designed to analyse the input images and then decide which images are real images and which images are fake images. The discriminator network uses convolution layers convolutional layers with a convolution kernel of 3, stride of 2, padding of 1, and the activation function of leaky ReLU is processed after every convolutional layer except for full connected layer which work to deliver an output. Softmax activation function is performed to compute the likelihood probability. This function is a function that turns a vector of K real values into a vector of K real values that sum to 1. The input value could be zero, positive or negative, but the softmax function transform them into values between 0 and 1. Mathematically, the sigmoid activation function is given by the following equation:

5 $$\sigma (z)=\frac{1}{1+{e}^{-z}}=\frac{{e}^{z}}{1+{e}^{z}}$$

$z$ is the input to the sigmoid function. It can be any real number, positive, negative, or zero. $\frac{1}{1+{e}^{-z}}$ calculates the final output of the sigmoid function. It takes the reciprocal of the denominator, resulting in a value between 0 and 1, which represents the output of the sigmoid activation function.

The generator was a U-Net including 5 blocks. The encoder and decoder structure allow the generator to extract comprehensive image features. The input of generator is a set of arbitrary numbers from the normal distribution, and its output is a fake image of shape 128 * 128 in RGB channel. The generator network has convolutional transpose layers. Every convolutional transpose layer is followed by and the activation function of leaky rectified linear unit (ReLU)^34^. The convolutional transpose layer converts a latent vector with 100 dimensions in latent space into a dense with size 128 * 128 * 3.

After feeding more than one thousand cell images belong to different classes to models, the pre-processing steps were performed to make the dataset more understandable for computer to train these networks. Data pre-processing in Machine Learning is a set of crucial steps that help enhance the quality of data to promote the extraction of meaningful insights from the data. The pre-processing steps include (1) Encoding the categorical data. In this paper, one-hot encoding was applied on each label to convert the categorical format into machine-understandable vectors. (2) Splitting the dataset. To split a dataset into a training set and a test set. (3) Feature scaling. It is a technique to standardize the independent features in the data in a fixed range.

### Swin transformer

Recently, transformers^35^ have greater domination of deep learning architecture than ever before in natural language processing (NLP) tasks. The triumph has motivate more research effort to adapt transformers for vision tasks. The Swin-transformer is one of the most exciting pieces of research following up from orginal ViT. Swin-transformer^36^ is a stand-of-art network which has transformer-based deep learning architecture with excellent performance in visions tasks. Compared to the Vision Transformer(ViT)^37^ that precedes it, Swin-transformer is highly efficent and more accurate. Thanks to these properties, Swin-transformer serves as the backbone in a lot of vision-based models today, it is a hierarchical Transformer model whose representation is compute with shift windows, which brings greater efficiency and allows for cross-window connection. Also, the hierarchical architecture brings flexibility to model at different scales and has linear computational complexity with respect to image size. The proposed method leverages the capabilities of the Swin-Transformer in processing microscopic images, effectively extracting image information for the detection of cell classes.

**Figure 8:**
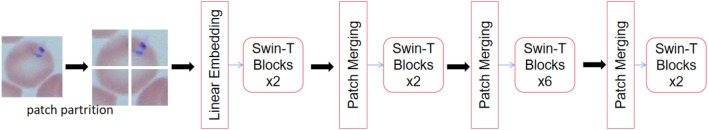
The architecture of Swin Transformer.

Figure 8 show the architecture of Swin-transformer(Swin-T). In Swin Transformer, the two key components are the ‘patch merging’ and the ‘Swin-T Block’.

**Figure 9:**
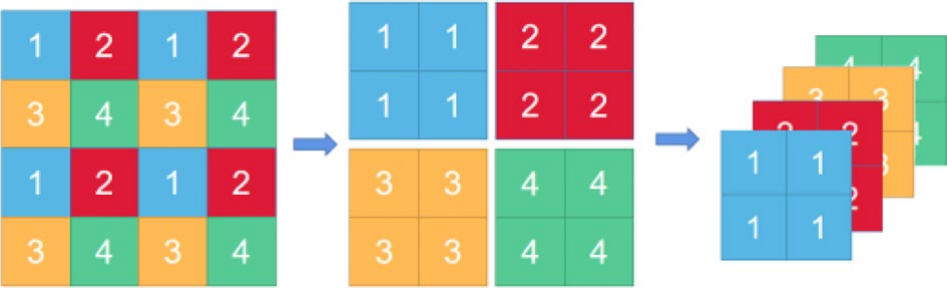
Patch merging. Due to a following 1 × 1 convolution, the final number of channels should be 2 × instead of 4 ×.

**Figure 10:**
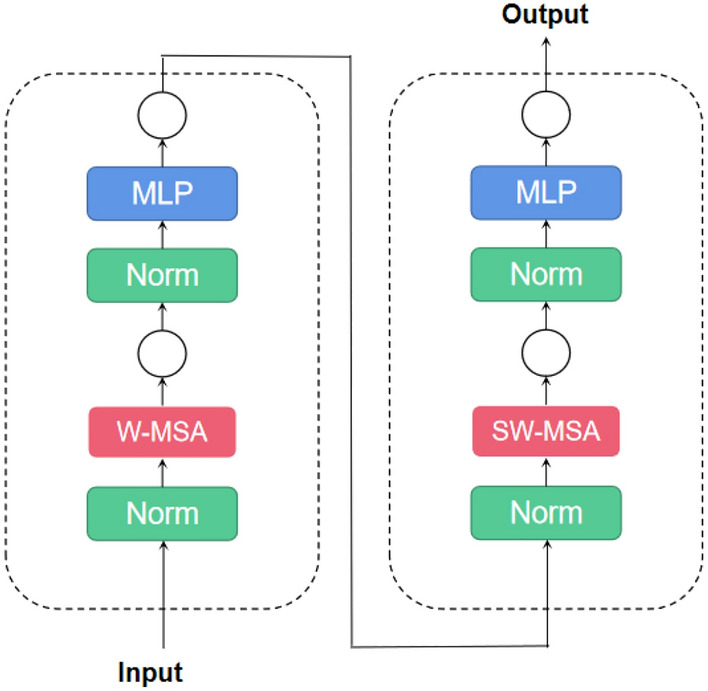
The Swin Transformer block with W-MSA and SW-MSA module.

As for the ‘patch merging’, the Swin Transformer builds herarchical feature maps by merging image patch as shown in Fig. 9, where ‘hierarchical’ refers to that the feature maps are merged from layer to layer, which is an effective downsampling operation reducing the spatial dimension of the feature maps from layer to layer. As for Swin-T block, it replace the standard multi-head self-attention(MSA) module in Vision Transformer with a Window-MSA and a Shifted Window MSA(SW-MSA) module. Figure 10 illustrate the Swin Transformer block. Moreover, in Swin-T block, the performance of SW-MSA is improved by using the masking technique and the relative positional encoding.

### Window-based self-attention

Window based Self-Attention(W-MSA) is proposed to compute self-attention within local windows. The W-MSA improve the efficiency of the model by arranging the windows to partition the images in a non-overlapping manner^36^.

The MSA in Vision Transformer computes the relationship between each patch against all other patches using global self-attention. As a result, it has a quadratic complexity with respect to the number of patches, leading to difficulty in processing high resolution images. The W-MSA, however, simply compute the self-attention only within each window that is a collection of patches. Supposing each window contain $\mathrm{M}*\mathrm{M}$ patches, the computational complexity of a global MSA module in ViT and a window-based approach on an image of $\mathrm{h}*\mathrm{w}$ patches are:

6 $$\begin{array}{c}\Omega (MSA)=4hw{C}^{2}+2(hw{)}^{2}C,\\ \Omega (W-MSA)=4hw{C}^{2}+2{M}^{2}hwC\end{array}$$

As evident from Eqs. (6), it is apparent that the computational complexity of W-MSA exhibits a linear relationship with $hw$ (the image size), whereas the computational complexity of MSA displays a quadratic relationship. When working with same image dimensions, the cost of windows is notably smaller compared to the cost of blocks. Consequently, this leads to a substantial reduction in computational complexity.

Due to the fixed window size throughout the whole network, the computational complexity of W-MSA is linear with respect to the number of patches or the resolution of the image, which greatly increases efficiency and saves computational resources compared to the quadratic complexity of standard MSA.

### Shifted window self-attention

Although the W-MSA has its merits, one obvious shortcoming is that the modelling power of the network was limited because the W-MSA restricts self-attention to each window and lacks connections across windows. To address this issue, the Shifted Window Self-Attention (SW-MSA) module is used after the W-MSA module to perform a shifted window partitioning approach.

Shifted Window MSA shifts the windows toward the bottom right corner by a factor of M/2.This Shift operation results in isolated patches not belong to any window, but Swin Transformer applied a ‘cyclic shift’ technique to move these patches into windows with incompete patches. Therefore, the windows may consist of patches not adjacent in the original feature map and a masking mechanism is employed to reduce self-attention computation to within each sub-window. This cyclic-shift introduces cross-connections between windows and improve the performance of the network while maintain the efficiency same as that of regular window partitioning.

### Light-weight vision transformer: MobileViT

ViT models for classification should be light-weight and quick to be effective. With regard to the performance on resource-constrained mobile devices, ViT models is much inferior to light-weight CNNs, even when the model size is reduced to fit the mobile devices with limited resource. DeiT(Data-Efficient Image Transformer)^38^, for example, is 3% less accurate than MobileNetv3^39^ due to a parameter budget of about 5–6 million. Therefore, designing a light-weight ViT model is a critical need. In this study, MobileViT played a pivotal role as a lightweight and rapid transformer model, efficiently handling cell images and extracting essential image data for cell class detection. Diverging from conventional transformer models with substantial parameter sizes, MobileViT boasts a reduced parameter count and demands fewer computing resources and memory, rendering it highly suitable for deployment on edge devices.

The MobileViT was proposed by by Sachin Mehta and Mohammad Rastegari in Ref.^28^. In contrast to ViT and its derivatives (with and without convolutions), it takes a different approach to learning global representations. For a standard convolution, the operation contains three steps: Unfolding, local processing, and folding. The MobileViT introduces a new layer to replace local processing in convolutions with global processing using transformers. Combining the strength of CNNs with transformers, this endows the MobileViT block both CNN and ViT-like features, simplifying its training steps while allowing it to learn better representations with fewer parameters. The most important attribute of the MobileViT is that it shows light-weight ViTs can achieve optimised computational performance in the level of light-weight CNN among a large number of mobile vision tasks using basic training methods.

### Ethics approval

The experimental protocol was established, according to the ethical guidelines of the Helsinki Declaration and was approved by the Human Ethics Committee of Xiangya Hospital. Written informed consent was obtained from individual or guardian participants.

## Results

Based on our experimental findings, it is evident that the Swin Transformer outperformed other models in terms of precision, recall, F1-score, specificity, and exhibited the lowest false positive rate (FPR). However, it is worth noting that the Swin Transformer lagged behind both MobileViT and MobileNet in terms of inference time, frames per second (FPS), and memory usage. In a holistic evaluation, the Swin Transformer and MobileViT emerged as standout performers among the state-of-the-art classifiers. Remarkably, MobileViT demonstrated the added advantage of being more resource-efficient, making it an ideal choice for edge devices with limited computing resources (On resource-constrained devices like smartphones, IoT devices, and edge devices, efficient inference is crucial. These devices often have limited computational power, so models must be optimized to run quickly).

The datasets we used for experiments consist of three sets: the original dataset that is taken from microscopic images, the augumented dataset that processed through rotation,cropping and other technices as well as the mixed dataset that we mixed fake images processed by WGAN-GP network with augmented dataset. The final dataset we used for model training is the mixed dataset. After we obtain the augmented dataset, the generator network was trained by looping over mini-batches of the augmented dataset to generate fake images and then the fake images were fed to the discrimitor to optimize the performance of the generator. This training could take lots of time to complete and may require many iterations to output good images. Finally, this strategy results in a generator that is able to generate convincingly realistic data and a discriminator that is able to learn strong feature representations of the real images. During this process, model loss fountions were introduced to evaluate the gradients of the discriminator and generator loss with respect to the learnable parameters of the discriminator and generator networks at each epoch, respectively. The loss in training generator and discriminator are determined as follows^27^:

7 $${\text{loss}}_{D} \, = \,\tilde{Y} - Y + \left( {\left\| {\nabla_{{\hat{X}}} \hat{Y}} \right\|_{2} - 1} \right)^{2} ,\,{\text{loss}}_{G} \, = \, - \tilde{Y},$$

where: ∇ denotes the gradient operator. Given an image $X$, a generated image $\widetilde{X}$, define $\widehat{X}=\varepsilon X$+(1-$\varepsilon )\widetilde{X}$ for random $\varepsilon \in$ *U*(0,1). $Y$, $\widetilde{Y}$ and $\widehat{Y}$ represent the output of the discriminator for the inputs $X$, $\widetilde{X}$ and $\widehat{X}$, respectively.

Table 2 shows the hyper-parameter configuration for WGAN-GP model during training process.

**Table 2:** Setting hyper-parameters for WGAN-GP model.

Name	Hyper-parameters
Input data	Augmented dataset
Image shape	128*128*3
Batch-size	64
Epochs	3000
Activation founction	LeakyReLU
Learning rate	0.0002
Optimizer	Adam
Clip value	0.01
Cpu thread	8
B1	0.5
B2	0.999

Hyperparameter tuning is an important and iterative process that requires several rounds of experimentation. Our objective is to strike a balance between exploration (trying different configurations) and exploitation (refining promising configurations). However, it's worth noting that WGAN-GP tends to converge more reliably, mitigating the risk of mode collapse—a common issue in GANs. Furthermore, the Adam optimizer typically demands less hyperparameter tuning when compared to SGD.

The Adam optimizer is known for its ability to adaptively adjust the learning rates for different model parameters, making it well-suited for various types of neural network architectures and training scenarios. In the Adam optimizer, "B1" and "B2" are hyperparameters that control the exponential moving averages of past gradients (first moment) and the squared gradients (second moment), respectively. These moving averages are used to adaptively adjust the learning rates for individual model parameters during optimization. Cross-entropy is used to measure the dissimilarity between predicted and true probability distributions. Minimizing this loss during training helps the model make accurate class predictions. In terms of activation founction, LeakyReLU helps mitigate this issue by allowing a small gradient for negative inputs, which keeps the neuron's learning alive.

**Figure 11:**
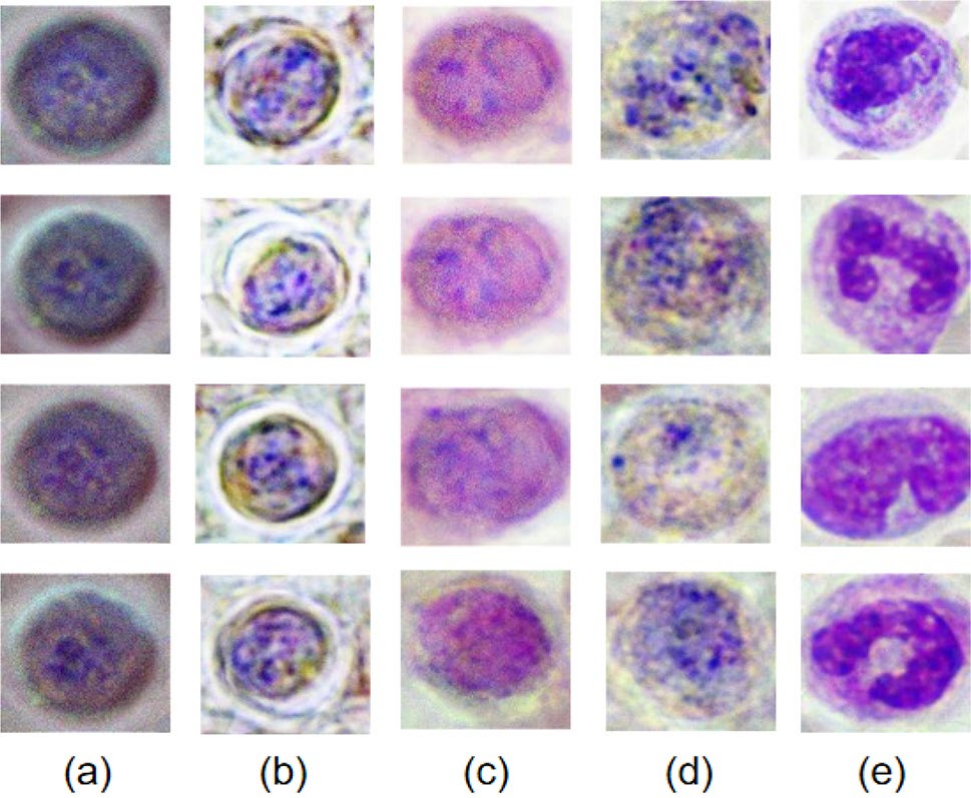
Fake images generate by WGAN-GP: (**a**) PF, (**b**) PM, (**c**) PO, (**d**) PV, (**e**) WBC.

After 3000 training epochs, we obtained the WGAN-GP model that generated 8400 fake cell images in each blood cell category for model training. The fake images belonging to different blood cell classes are shown in Fig. 11. Those images were then combined with augmented dataset and randomly spilt into three subsets (training set, valiadation set and testing set) by the ratio of 13:4:3.

In summary, after collecting 2061 images from the original microscopic dataset, we expanded it to create a dataset comprising 14,400 images through the use of augmentation techniques. Furthermore, we carried out additional augmentation to yield an extensive dataset totaling 64,800 images using WGAN-GP. Table 3 presents the Details of the dataset in three different periods. The final testing set consists of six classes and each cell class contains 1620 images.

**Table 3:** Data distribution during different periods.

Blood cell catogories	Original dataset	Augumented dataset	Mixed dataset
Plasmodium falciparum	187	2400	10,800
Platelet	457	2400	10,800
Plasmodium malariae	113	2400	10,800
Plasmodium ovale	93	2400	10,800
Plasmodium vivax	168	2400	10,800
White blood cells	300	2400	10,800
Red blood cells	300	2400	10,800
Total	2061	14,400	64,800

**Figure 12:**
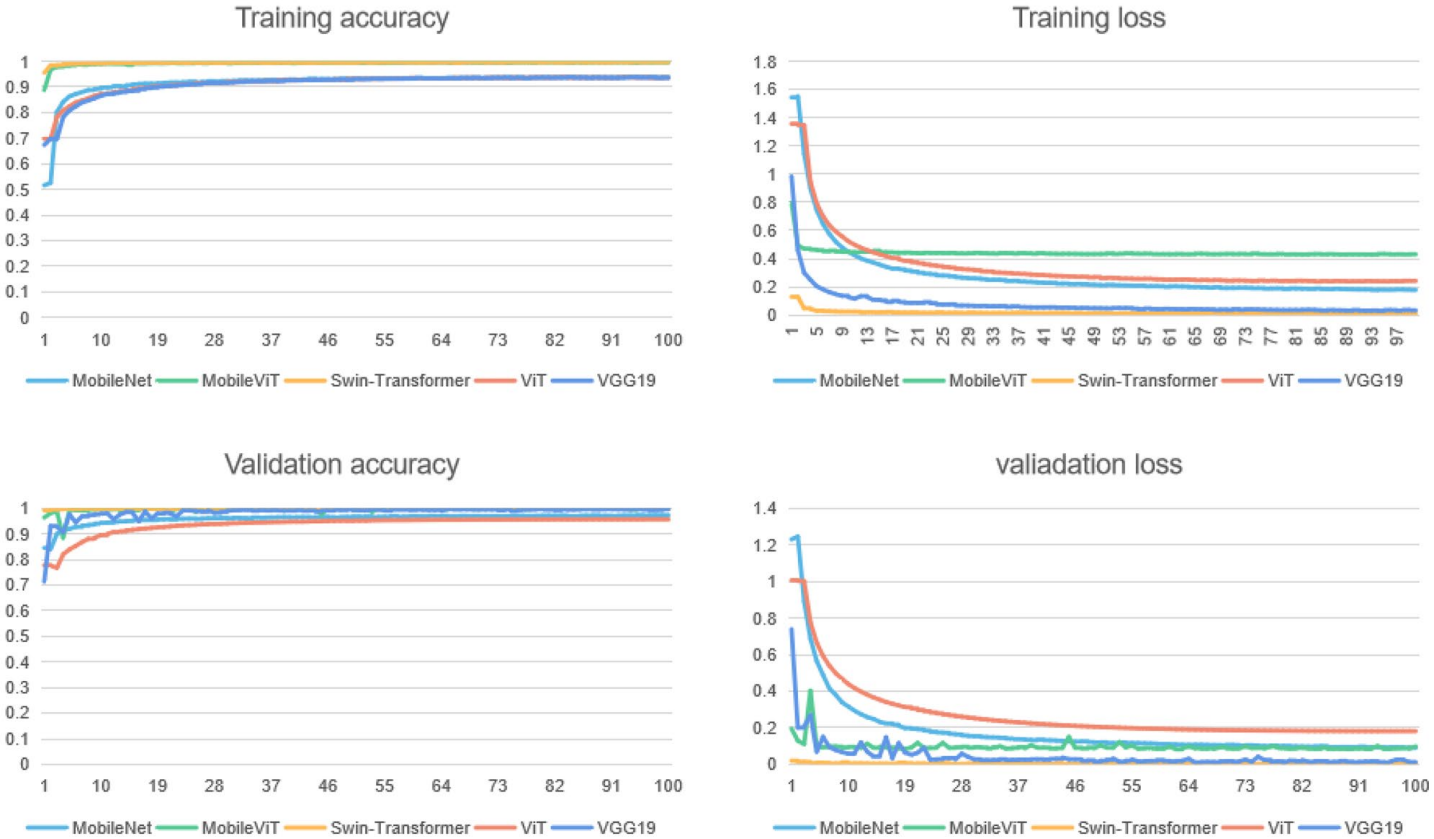
The performance of four different network after the training process with mixed malaria cell image dataset.

The batch size is configured to be either 64 or 128, depending on the availability of GPU memory. For our loss function, we have opted for CrossEntropyLoss. The training process spans a total of 100 epochs. Learning curves are plots that show changes in learning performance over time in terms of experience as shown in Fig. 12. Those curves of different models represent their performance on the train and validation datasets can be used to diagnose an underfit, overfit, or well-fit model. The Swin Transformer and the MobileViT exhibited remarkable accuracy curves and smooth loss curves compared to other architectures, firmly establishing their superiority among the state-of-the-art deep learning models.

The detailed architecture analysis of the Swin Transformer, Mobile-ViT and other deep learning architecture with AdmaW optimizer and weight decay is outlined in Table 4. Obviously, Swin-Transformer outperforms other methods in Precision, recall, F1-score and specificity. Table 5 states the prediction accuracy of each model architecture at each blood cell class. The maximum average accuracy was 99.885% with Swin Transformer. On the other hand the MobileNet has the least inference time as shown in Table 6. However, to balance both inference speed and accuracy, MobileViT should be the best choice.

**Table 4:** Precision, Recall, F1-score and Specificity in percent (%) for different trained deep learning models.

	Precision	Recall	F1-score	Specificity	FPR
Swin-T	99.885	99.316	99.599	99.981	0.019
ViT	86.526	51.697	64.723	97.470	2.530
Mobile-ViT	99.868	99.212	99.539	99.978	0.022
VGG-19	79.489	39.242	52.544	95.877	4.123
MobileNet	96.570	82.431	88.942	99.411	0.589

**Table 5:** Accuracy in percent for different networks on unseen data.

	PF	PLT	PM	PO	PV	RBC	WBC	Average
Swin-T	99.815	99.815	99.938	99.877	99.877	99.877	100.000	99.885
ViT	88.025	90.802	89.198	74.938	66.543	98.827	97.346	84.722
Mobile-ViT	99.753	100.000	99.938	99.753	99.815	99.815	100.000	99.867
VGG-19	74.568	48.148	99.444	76.235	78.457	79.568	100.000	79.488
MobileNet	96.049	94.877	99.012	99.012	92.346	95.000	99.444	96.534

**Table 6:** Inference time, Frames per Second and Memory size of Model architecture.

Network	Inference time (ms)	FPS	Memory (MB)
Swin-T	20.070	49.825	107.81
ViT	32.422	30.842	335.22
Mobile-ViT	16.463	60.742	3.85
VGG-19	23.565	42.436	545.32
MobileNet	10.322	96.878	8.96

In summary, the Swin Transform deliver better accuracy compared to other neural networks in terms of malaria-infected microscopic blood cell classification, and MobileViT achieve almost the same efficacy with lower memory usage and faster inference time. In our deep learning models, an inference means the forward propagation process, which given a blood cell image, gets a classification result. This classification result determines the class of the cell image. Knowing the inference time in advance can help you design a model that will perform better, and be optimized for inference.

Table 6 demonstrates that MobileNet and Mobile-ViT outperformed other models in terms of short inference times, high frames per second (FPS), and low memory utilization when applied to blood cell images captured from smeared blood samples. This shows their potential for deployment on edge devices or smartphones characterized by low memories and limited computational resources.

## Discussions

Our work has certain limitations. Firstly, our approach has not been assessed on extensive multiclass datasets, primarily due to the limited number of patients available for this study. However, it is worth noting that previous experiments involving transformers have been conducted on large binary datasets^16,40^. Secondly, we have refrained from testing larger models with an increased number of parameters in this research, primarily due to the requirement for more powerful GPU resources.

According to our results, swin-transformer outperform the baseline architectures in terms of precision, recall, F1-score, specificity, and FPR on test set. There are several reasons: (1)Hierarchical Structure: Swin Transformer introduces a hierarchical structure that breaks down the input image into a series of smaller non-overlapping patches. (2) Shifted Windows: This shift operation helps the model attend to neighboring regions, improving its ability to capture spatial relationships and reducing the risk of information loss at patch boundaries. (3) Local–Global Attention: Swin Transformer incorporates both local and global attention mechanisms. The local attention mechanism enables the model to focus on nearby patches.

It's worth noting that the performance of a model can vary depending on the specific task, dataset, and the quality of training and fine-tuning. While Swin Transformer has shown promising results in our experiment, it may not always be the best choice for every computer vision task. For edge devices, MobileViT with less parameters is a more suitable approach to achieve the balance between high accuracy and low resource consumption.

Table 7 shows the demonstrates the detailed comparisons between existing and the proposed methods.

**Table 7:** Previous deep learning methodologies for malaria classification.

Reference	Dataset	Methods	Results	Advantage	Limitations
^16^	27,558 images with equal instances of parasitized and uninfected cells	MobileViT	accuracy of 98.37%	Stability to preventing biasedness	oscillation of accuracy and loss curves
^40^	27,558 images with equal instances of parasitized and uninfected cells	compact convolutional transformer	accuracy of 99.23%	CCT with Grad-CAM visualization	No comparison with other models
^41^	27,558 images with equal instances of parasitized and uninfected cells	Cycle GAN with dynamic criterion	FID score of 0.0043	high quality synthetic blood cell images	Only two cell classes were tested
^21^	27,558 images with equal instances of parasitized and uninfected cells	MPP algorithm for edge-based segmentation	F-measure values of 0.9	high accuracy	Lack of the classification part
^26^	345 images consisting of 111 blood cells on average	A two-stage approach	Average accuracy of 96.26%	a userfriendly mobile-based application is built	Need to employing the mobile app on a large scale to verify its efficacy
Ours	2061 images with 6 classes of cells	Transformers and WGAN-GP	A balance between high accuracy and low resources consumption	Combination of augmentation techniques and transformers	Lack of a multiclass large dataset

## Conclusion

Malaria, a severe febrile condition brought about by Plasmodium parasites, results in tens of thousands of fatalities annually. The traditional method of malaria diagnosis, involving the microscopic examination of stained blood slides, is favored for its affordability and accessibility. Nevertheless, this procedure poses significant challenges, as it necessitates a proficient workforce of medical laboratory technicians—a resource that is both valuable and scarce on a global scale.

In this paper, we developed a deep learning-based automated mechanism to help and assist the doctors and patients in malaria parasite screening at its early stage. A new dataset of microscopic images of blood cells and different plasmodium species was collected, labelled and then augmented with WGAN-GP and other techniques. The mean accuracy of the MobileViT and Swin Transformer were 99.885% and 99.867% respectively.

Our results unequivocally validate the effectiveness and efficiency of the chosen models for multiclass Plasmodium classification. Notably, both the Swin Transformer and MobileViT surpass traditional CNNs in performance, with MobileViT being particularly well-suited for edge devices with constrained computational capabilities. Moreover, the incorporation of the WGAN-GP and other augmentation techniques proves to be valuable tools for expanding the image dataset.

The research's clinical relevance lies in its potential to revolutionize malaria diagnosis by providing accurate, cost-effective, and accessible solutions. This could lead to earlier detection, improved patient outcomes, and a more efficient allocation of healthcare resources, ultimately contributing to the global efforts to control and eliminate malaria. Enhancing the sensitivity of the malaria diagnosis system will not only improve its accuracy but also enable it to tackle more intricate diagnostic challenges. This can lead to earlier and more precise diagnoses, ultimately benefiting both individual patients and public health efforts to control and eradicate malaria.

In our future work, we intend to cultivate a heightened sensitivity within our system, enabling it to adeptly tackle intricate tasks like parasite number counting, the analysis of ambiguously smeared image slices, and the multi-stage lifecycle classification of Plasmodium. To achieve this objective, we will implement several strategies: (1) Lager Dataset: We will expand the diversity and size of our training dataset by enlisting the participation of more volunteers and implementing a broader range of data augmentation techniques. This approach will empower our system with a more extensive and nuanced understanding of malaria-related image data. (2) Advanced Transfer Learning: Building upon our existing utilization of transfer learning techniques, we will remain vigilant for improved pre-trained models that become available. Leveraging these models, which have been honed on expansive microscopy image datasets, will significantly elevate the sensitivity of our system, enhancing its ability to discern intricate patterns and features. (3) Integration of Multiple Data Sources: We will explore the integration of multiple data sources beyond image analysis. Incorporating supplementary information such as patient demographics, geographic data, and climate data will infuse our system with a holistic perspective. This multi-source data fusion will bolster the system's sensitivity, empowering it to offer more accurate predictions regarding malaria cases and their potential severity.

We have assembled a novel multiclass dataset encompassing PLT, WBC, RBC and four malaria species, namely PO, PV, PF, and PM. This dataset serves as a pivotal resource for training deep learning-based research dedicated to addressing this challenging problem. To tackle the inherent imbalance within the dataset, we introduced a Generative Adversarial Network (GAN) as a strategic solution. Furthermore, our investigation revealed the exceptional efficiency of two transformer models in the context of multiclass malaria parasite classification.

## Data Availability

The data are not publicly available due to privacy. The data presented in this study are available on request from the corresponding author.
