# Author Correction: Multiclass malaria parasite recognition based on transformer models and a generative adversarial network

**DOI:** 10.1038/s41598-023-45535-z

**Published:** 2023-10-24

**Authors:** Dianhuan Tan, Xianghui Liang

**Affiliations:** Department of Clinical Laboratory, Xiangya Hospital, Central South University, No.87 Xiangya Road, Kaifu District, Changsha, 410008 Hunan China

Correction to: *Scientific Reports* 10.1038/s41598-023-44297-y, published online 10 October 2023

The original version of this Article contained an error in Affiliation 1, which was incorrectly given as ‘Xiangya Hospital, Changsha, China’.

The correct affiliation is listed below.

Department of Clinical Laboratory, Xiangya Hospital, Central South University, No.87 Xiangya Road, Kaifu District, Changsha, 410008, Hunan, China.

The original Article has been corrected.

